# An assessment of the extent to which the contents of PROSPERO records meet the systematic review protocol reporting items in PRISMA-P

**DOI:** 10.12688/f1000research.25181.2

**Published:** 2020-09-10

**Authors:** Alison Booth, Alex S. Mitchell, Andrew Mott, Sophie James, Sarah Cockayne, Samantha Gascoyne, Catriona McDaid

**Affiliations:** 1 Department of Health Sciences, York Trials Unit, University of York, York, YO10 5DD, UK

**Keywords:** Systematic review, protocol, reporting, registration

## Abstract

**Background:** PROSPERO is an international prospective register for systematic review protocols. Many of the registrations are the only available source of information about planned methods. This study investigated the extent to which records in PROSPERO contained the Preferred Reporting Items for Systematic Review and Meta-Analysis Protocols (PRISMA-P).

**Methods**: A random sample of 439 single entry PROSPERO records of reviews of health interventions registered in 2018 was identified. Using a piloted list of 19 PRISMA-P items, divided into 63 elements, two researchers independently assessed the registration records. Where the information was present or not applicable to the review, a score of 1 was assigned. Overall scores were calculated and comparisons made by stage of review at registration, whether or not a meta-analysis was planned and whether or not funding/sponsorship was reported.

**Results**: Some key methodological details, such as eligibility criteria, were relatively frequently reported, but much of the information recommended in PRISMA-P was not stated in PROSPERO registrations. Considering the 19 items, the mean score was 4.8 (SD 1.8; median 4; range 2-11) and across all the assessed records only 25% (2081/8227) of the items were scored as reported. Considering the 63 elements, the mean score was 33.4 (SD 5.8; median 33; range 18-47) and overall, 53% (14,469/27,279) of the elements were assessed as reported. Reporting was more frequent for items required in PROSPERO than optional items. The planned comparisons showed no meaningful differences between groups.

**Conclusions**: PROSPERO provides reviewers with the opportunity to be transparent in their planned methods and demonstrate efforts to reduce bias. However, where the PROSPERO record is the only available source of *a priori* reporting, there is a significant shortfall in the items reported, compared to those recommended. This presents challenges in interpretation for those wishing to assess the validity of the final review.

## Introduction

Detailing the planned methods for conducting a systematic review in advance of commencing the review is essential in order to minimise a range of potential biases ^1, 2^. The plan, set out in a protocol, should ideally be made available in the public domain to facilitate transparency ^3, 4^. In addition, registration of key protocol details is encouraged as best practice in reporting guidelines ^5, 6^ by publishers like the British Medical Journal (BMJ), Public Library of Science (PLoS), and BioMed Central (BMC), and is mandated in their instructions to authors by journals such as BMC Systematic Reviews, BMJ, BMJ Open, PLoS One, and National Institute for Health Research (NIHR) journals.

There are a number of options for putting systematic review protocols into the public domain, such as publication in open access journals like BMC Systematic Reviews and uploading to open data repositories like the Open Science Framework (OSF) ( https://osf.io/registries/discover?q=protocols). PROSPERO ( https://www.crd.york.ac.uk/prospero/) is a facility for registering key methodological details in advance of carrying out a review. Registration on PROSPERO requires completion of an internationally agreed minimum dataset for a systematic review protocol ^7, 8^. Registrants also have the option of uploading their protocol or providing a hyperlink to it.

PROSPERO remains the only free, open access registry of systematic review protocols, making it a single searchable source of the protocols of on-going and completed reviews. Uptake of registration has increased exponentially and by the end of 2019 there were over 60,000 registrations in PROSPERO. There is evidence that considerably more systematic reviews are registered in PROSPERO than have peer-reviewed protocols published. In 2016, 1058 records were accepted by PROSPERO; in the same time period, only 404 published systematic review protocols were identified ^3^. Another study reported identifying 20,814 non-Cochrane systematic review protocols from web scraping PROSPERO and bibliographic database searches. Of these, 924 were only published in journals, 807 were published in journals and registered in PROSPERO and 19,890 were only available as a record in PROSPERO ^9^. There is further evidence from Ge *et al*. (2018) that of the non-Cochrane reviews registered in PROSPERO, only 3% or 4% have a published protocol ^9, 10^. This means that for a large number of reviews a PROSPERO record is likely to be the only source providing details of the planned methods.

Published protocols and registration records aim to provide transparency in the review process by allowing public access to the key pre-specified elements for the conduct of a review. One of the stated aims of PROSPERO is to facilitate comparison between planned review methods and reported results ^8^. Such a comparison enables peer reviewers and other readers of the final review to assess for themselves the potential for bias in the findings. There is also a steadily growing body of research using PROSPERO records to assess the risk of biases in final review reports ^10– 15^. Given this reliance on the information provided in PROSPERO records, it is important to understand the level of detail provided in records. The focus of this study was on the stated aim of PROSPERO to reduce the opportunity for bias by enabling comparison of the completed review with what was planned in the protocol ^8^.

The Preferred Reporting Items for Systematic Reviews and Meta-Analyses extension for Protocols (PRISMA-P) were developed through expert consensus using internationally compiled datasets such as PROSPERO and SPIRIT ^4, 6^.

Key methodological aspects of a protocol are mandated for registration in PROSPERO; other items, mainly administrative fields, are optional ^7, 8^. Submissions for registration are not subject to any form of peer review or critical appraisal, they are simply checked for sense but not methodological rigor. Therefore, there is the possibility that PROSPERO records do not provide all the necessary information identified by the PRISMA-P guidelines to enable comparison with the completed systematic review. The registration record may be the only place where *a priori* methods are available for users, in particular peer reviewers, to check for potential issues such as selection, outcome reporting and publication biases. This study investigated the extent to which records in PROSPERO, where no protocol or other information was available, comply with each of the items for reporting of protocols set out in the PRISMA-P guidelines.

## Methods

A random sample of PROSPERO registration records were assessed against the systematic review protocol reporting criteria set out in the PRISMA-P 2015 checklist ^4^. Key methods are provided here with further details available in the protocol for this study, which was prepared and made publicly available on the OSF, 17 March 2020 ( *Extended data* ^16^).

### Study sample of PROSPERO records

A dataset of non-Cochrane PROSPERO records was provided by Metaxis, the software managers of PROSPERO. Records of reviews defined by the record holder as a health intervention registered on or between 1 January 2018 and 31 December 2018, were identified.

Cochrane reviews, reviews of animal studies, non-intervention reviews as identified in PROSPERO, i.e. Diagnostic accuracy, Prognostic factors, Prevention, Epidemiological reviews relevant to health and social care, Public health, Service delivery in health and social care, Methodological reviews, reviews of reviews, and synthesis of qualitative studies, were all excluded as PROSPERO and PRISMA-P were developed for reviews of interventions. Only records with no evidence from the registration record of other protocol related information, for example in a published protocol or other links in the PROSPERO record, were included and we restricted the data set to those records with a single registry entry.

Records from the calendar year 2018 were used to allow time for dissemination and adoption of the PRISMA-P guidelines published in 2015. A sample of 20% of these records was randomly selected using simple random sampling for assessment against the PRISMA-P reporting criteria.

### Assessment tool and scoring

The PRISMA-P checklist recommends 17 numbered items, with nine subdivisions, totalling 26 items be reported in a systematic review protocol ^4^. Seven of the 26 items were excluded from the assessment as they would always or never meet registration requirements in PROSPERO. For example, registration is implicit for a record accepted in PROSPERO, and there is no field for author contributions or sponsor role so these would never be reported. The study assessment tool, developed specifically for this study as a Google Form, therefore contained 19 of the PRISMA-P items. Where the PRISMA-P description for an item specified more than one piece of information, the individual elements were listed as subsets of the items ^4, 6^. For example, item 14. Risk of bias in individual studies, says: “Describe anticipated methods for assessing risk of bias of individual studies, including whether this will be done at the outcome or study level, or both; state how this information will be used in data synthesis.” Scoring for this item was for each of the following separate elements: No risk of bias assessment planned and justification provided; Risk of bias tools named for all study types included; Outcome or study level or both; Domains/outcomes for risk of bias assessment stated; Risk of bias assessment process described; How risk of bias findings will be used in synthesis. Applying this approach to the 19 items resulted in a list containing 63 elements to be reported.

Where an item was reported or not applicable, a score of 1 was assigned. Where the information was not reported this scored 0. The maximum possible overall score for the PRISMA-P listed items was 19 per record. Scores for the breakdown of individual elements within the items was also reported, the maximum possible score was 63 per record.

### Assessment procedure

The researchers undertaking the assessments (AB, ASM, AM, SJ, SC, SG) familiarised themselves with both PRISMA-P papers ^4, 6^. All had previously received training in systematic review methods and/or authored at least one systematic review. The draft assessment form and accompanying guidance notes were revised and finalised during a training session and piloted with the aim of achieving greater than 90% agreement.

Two researchers independently compared the information provided in each PROSPERO record with the relevant items in the study assessment tool. Options for decisions were: Reported (information provided as per PRISMA-P requirements); Not reported (some or all information not provided); and, Not applicable (where an item was not relevant to an individual record, e.g. a meta-analysis was not planned).

Records were randomly assigned to assessors by first creating a list of the sampled record unique identification numbers and dividing the list into 14 blocks of approximately equal size, with each block being assigned a colour. A copy of this list together with the block configuration was then placed alongside the original list. Seven sub-lists were then created by randomly selecting a block from the first list and a block from the second list, such that blocks of the same colour were not in the same sub-list, and each colour appeared in two sub-lists. Each sub-list was then randomly assigned to an assessor.

It was not feasible to blind the researchers to the authors of registrations in PROSPERO. None of the assessors were authors of included registrations. On completion of the pilot assessments and the full set of records, disagreements were resolved through discussion or recourse to a third researcher.

The assessment form and the guidance notes are available on the OSF ( *Extended data* ^16^).

### Analysis

The primary outcome for this study was the compliance of PROSPERO registration records to PRISMA-P reporting items. This was measured by the total mean score allocated by the two independent assessors to each of the 19 items assessed (maximum possible score 19) for each record and by the total mean score for the individual elements within items (maximum possible score 63). Overall scores for the assessed dataset, scores by the 19 PRISMA-P items and by the 63 elements were the planned outcome measures.

For the eligible 2018 records that were assessed and those not assessed, demographic data for month of registration, funding/sponsor, planned meta-analysis, number of authors, stage of review at registration, topic and country of review were to be reported. Comparisons to identify any association between records registered before or after screening started; whether a meta-analysis was planned or not; and whether a review was funded/sponsored or not and completeness of reporting of items were planned.

### Deviations from protocol

During piloting of the assessment form, it became clear that it would not be possible to assess records for PRISMA-P item 5a Sources and 5b Sponsor. This would have required separating sources of financial support from sponsorship or any other form of support as reported in the single PROSPERO field, which was not possible. This item was therefore removed from the assessment form. Instead, a series of regular expression patterns was compared to the list of eligible records to identify those where the record contained any indication of funding/ sponsorship/support or indicated there was none. These data were used in the presentation of demographics and subgroup comparison.

## Results

**Figure 1.**
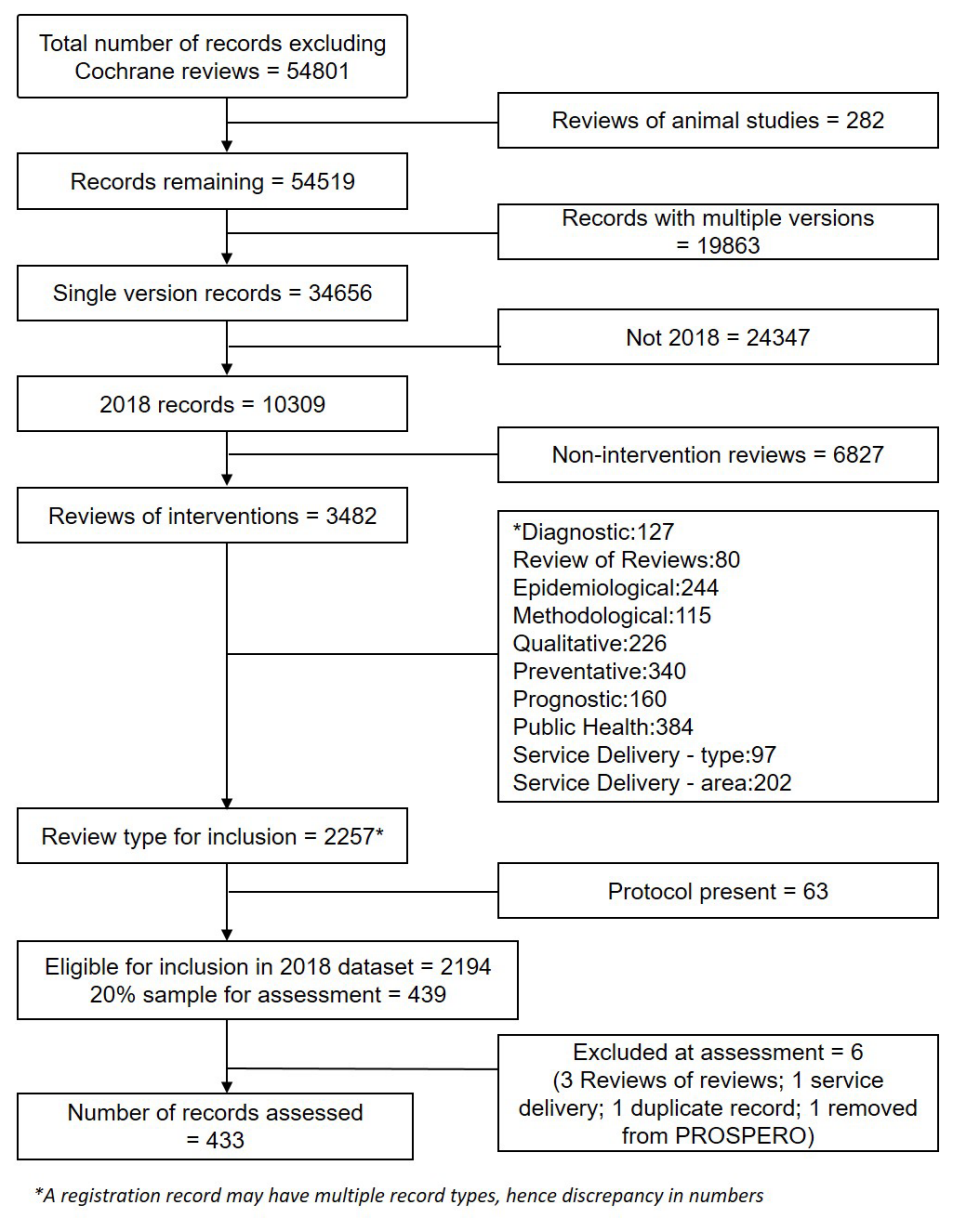
Flow chart of record sample identification.

The PROSPERO dataset contained 5,313 records for reviews of health interventions first accepted in 2018 (excluding Cochrane and reviews of animal studies). Applying the other study inclusion/exclusion criteria resulted in 2,194 eligible registration records. The randomly selected sample of 20% for assessment included 439 records. During assessment, six records were excluded, for not meeting the inclusion criteria (4), being a duplicate (1) or no longer available on PROSPERO (1). Assessments were therefore carried out on 433 PROSPERO records. A flow chart of record selection is shown in Figure 1.

Agreement following initial piloting of the assessment form was 87%; after further discussions and revision of the assessment guidance notes and form a second pilot achieved 92% agreement. For all the records assessed, agreement between researchers was 90%, all differences were resolved through discussion or referral to a third researcher.

Demographic details of the sample of PROSPERO records selected for assessment and those not assessed are provided in Table 1. The number of authors listed ranged between one and 17, with the exception of a single record, included in the assessed sample, where 47 authors were listed. The eligible sample for 2018 included records from 67 different countries: 20 records listed two countries and 15 listed between three and nine countries involved in the review. There were no substantial differences between the data sets in the month of registration; whether any details of funding and/or sponsorship were provided; whether a meta-analysis was planned or not; the number of authors listed per record; stage of review at registration; topic of review or country involved in undertaking the review.

**Table 1.** Demographic details of non-sample set and sample set of the eligible 2018 PROSPERO records.

Demographic		Records for assessment (n = 439)	Records not assessed (n = 1755)
**Month of registration n (%)**	*January*	45 (10)	168 (10)
	*February*	32 (7)	141 (8)
	*March*	25 (6)	100 (6)
	*April*	35 (8)	122 (7)
	*May*	16 (4)	110 (6)
	*June*	36 (8)	151 (8)
	*July*	54 (12)	188 (11)
	*August*	56(12)	200 (11)
	*September*	31 (7)	151 (9)
	*October*	37 (8)	138 (8)
	*November*	37 (8)	160 (9)
	*December*	35 (8)	126 (7)
**Funding/support indicated n (%)**		386 (88)	1572 (90)
**Meta-analysis planned n (%)**		253 (58)	1064 (61)
**Number of listed authors (mean, range)**		4.1 (0 – 47 *)	3.9 (0 – 17)
Stage of review ** **n (%)**	*Not Started*	96 (22)	385 (22)
	*Searches Start*	65(15)	283 (16)
	*Searches Complete*	12 (3)	57 (3)
	*Pilot Selection Start*	56 (13)	252 (14)
	*Pilot Selection Complete*	16 (4)	50 (3)
	*Screening Start*	80 (19)	285 (16)
	*Screening Complete*	13 (3)	56 (3)
	*Extraction Start*	93 (21)	376 (21)
	*Extraction complete*	2 (0)	8 (1)
Topic of review *** **n (%)**	*Alcohol/substance misuse/* *abuse*	12 (3)	28 (2)
	*Blood and immune system*	13 (3)	90 (5)
	*Cancer*	42 (10)	182 (10)
	*Cardiovascular*	61 (14)	220 (13)
	*Care of the elderly*	16 (4)	72 (4)
	*Child health*	31 (7)	139 (8)
	*Complementary therapies*	43 (10)	178 (10)
	*Crime and justice*	0 (0)	2 (0)
	*Dental*	30 (7)	138 (8)
	*Digestive system*	34 8)	127 (7)
	*Ear, nose and throat*	7 (2)	27 (2)
	*Education*	10 (2)	23 (1)
	*Endocrine and metabolic* *disorders*	35 (8)	144 (8)
	*Eye disorders*	3 (1)	16 (1)
	*General interest*	5 (1)	29 (2)
	*Genetics*	3 (1)	5 (0)
	*Health inequalities/health* *equity*	3 (1)	8 (1)
	*Infections and infestations*	22 (5)	97 (6)
	*International development*	0 (0)	2 (0)
	*Mental health and* *behavioural conditions*	51 (12)	129 (7)
	*Musculoskeletal*	70 (16)	253 (14)
	*Neurological*	44 (10)	208 (12)
	*Nursing*	11 (3)	45 (3)
	*Obstetrics and gynaecology*	23 (5)	101 (6)
	*Oral health*	21 (5)	100 (6)
	*Palliative*	4 (1)	16 (1)
	*Perioperative care*	14 (3)	81 (5)
	*Physiotherapy*	36 (8)	129 (7)
	*Pregnancy and childbirth*	13 (3)	60 (3)
	*Public Health*	0 (0)	0 (0)
	*Rehabilitation*	43 (10)	173 (10)
	*Respiratory disorders*	16 (4)	87 (5)
	*Service delivery*	0 (0)	0 (0)
	*Skin disorders*	12 (3)	40 (2)
	*Social care*	0 (0)	2 (0)
	*Surgery*	49 (11)	209 (12)
	*Tropical medicine*	0 (0)	0 (0)
	*Urological*	20 (5)	71 (4)
	*Wounds, injuries and* *accidents*	11 (3)	70 (4)
	*Violence and abuse*	3 (1)	10 (1)
Country of review *** **n (%)**	*Australia*	33 (8)	143 (8)
	*Brazil*	53 (12)	224 (13)
	*Canada*	38 (9)	121 (7)
	*China*	100 (23)	414 (24)
	*England*	46 (10)	163 (9)
	*Germany*	13 (3)	40 (2)
	*Italy*	14 (3)	62 (4)
	*Netherlands*	13 (3)	51 (3)
	*Spain*	13 (3)	39 (2)
	*USA*	48 (11)	160 (9)
	*57 other countries*	127 (29)	562 (32)

* the record with 47 authors was a single outlier: range excluding this record was 0–15 ** details for three records were not available on PROSPERO *** all items reported by authors included; therefore totals are more than the number of records

None of the PROSPERO records assessed against the eligibility criteria reported on all elements in each of the items recommended for a systematic review protocol in the PRISMA-P guidelines. The mean total score for individual PROSPERO records, where 1 point was gained for each of the 19 items in the PRISMA-P checklist, was 4.8, the standard deviation 1.8, the median 4, and range 2 to 11. Considering all items across all the assessed records, only 25% (2081/8227) of the items were scored as reported.

The mean total score for individual PROSPERO records where 1 point was gained for each of the 63 elements of the PRISMA-P reporting guidelines was 33.4, the standard deviation 5.8, the median 33 and the range 18–47. Overall, 53% (14,469/27,279) of the elements were considered as reported.

### Scoring for 19 PRISMA-P items

The highest scoring item was PRISMA-P 1b which requires the protocol to be identified as to whether it is an update of a review; the high score was the result of this being a not-applicable item for 423 (98%) of the 433 records ( Table 2). Eligibility criteria (study design, setting, population, intervention, comparator, outcomes) was the next highest scoring item with 386 (89%) reporting all of these elements. Selection process (214, 49%), describing the criteria under which study data will be quantitatively synthesized (200, 46%), and describing the type of summary planned if quantitative synthesis is not appropriate (227, 52%) were the next highest scoring of the 19 items assessed.

**Table 2.** Assessment scores by item and breakdown for 433 PROSPERO records.

PRISMA-P reporting item	Reported or not applicable n (%)	Not reported n (%)	Breakdown of items	Reported n (%)	Not reported n (%)	Not applicable n (%)
Section 1 Administrative information						
1a. Identification in the title: Identify the report as a protocol of a systematic review	22 (5)	411 (95)	Identify the report as a protocol	22 (5)	411 (95)	/
			Identify the report as a systematic review	342 (79)	91 (21)	/
1b. Update: If the protocol is for an update of a previous systematic review	424 (98)	9 (2)	Identify the report as an update	1 (0)	9 (2)	423 (98)
Section 2 Introduction						
6. Rationale: Describe the rationale for the review in the context of what is already known	38 (9)	395 (91)	Rationale described	44 (10)	389 (90)	/
			Context provided *	108 (25)	325 (75)	/
7. Objectives: Provide an explicit statement of the question(s) the review will address with reference to participants, interventions, comparators, and outcomes (PICO) *	134 (31)	299 (69)	Population	397 (92)	36 (8)	/
			Intervention	416 (96)	17 (4)	/
			Comparator	142 (33)	264 (61)	27 (6)
			Outcomes	237 (55)	196 (45)	/
Section 3 Methods						
8. Eligibility criteria: Specify the study characteristics (e.g., PICO, study design, setting, time frame) and report characteristics (e.g., years considered, language, publication status) to be used as criteria for eligibility for the review *	386 (89)	47 (11)	Study design specified *	427 (99)	6 (1)	/
			Setting (condition or domain) specified *	410 (95)	23 (5)	/
			Population *	429 (99)	4 (1)	/
			Intervention *	428 (99)	5 (1)	/
			Comparator *	392 (91)	14 (3)	27 (6)
			Outcome(s) *	424 (98)	9 (2)	/
9. Information sources: Describe all intended information sources (e.g., electronic databases, contact with study authors, trial registers, or other grey literature sources) with planned dates of coverage *	2 (1)	431 (99)	Electronic database(s) named	431 (99)	2 (1)	/
			Grey literature sources	100 (23)	333 (77)	/
			Study registries	289 (67)	144 (33)	/
			Contact with study authors planned or statement that contact not planned	27 (6)	406 (94)	/
			Other: e.g. hand searching reference lists of included studies	152 (35)	281 (65)	/
			Planned search dates	238 (55)	195 (45)	/
10. Search strategy: Present draft of search strategy to be used for at least one electronic database, including planned limits, such that it could be repeated	75 (17)	358 (83)	Draft search strategy provided	91 (21)	342 (79)	/
			Search terms given alone	100 (23)	242 (56)	91 (21)
			Approach to limits/ restrictions reported e.g. language or dates/ statement of no limits *	332 (77)	101 (23)	/
11a. Data management: Describe the mechanism(s) that will be used to manage records and data throughout the review	17 (4)	416 (96)	Software named/type indicated *	56 (13)	377 (87)	/
			De-duplication planned	42 (9)	391 (91)	/
11b. Selection process: State the process that will be used for selecting studies (e.g., two independent reviewers) through each phase of the review (i.e., screening, eligibility, and inclusion in meta-analysis)	214 (49)	219 (51)	Initial screening process described *	232 (54)	201 (46)	/
			Full paper screening process described *	219 (51)	214 (49)	/
11c. Data collection process: Describe planned method of extracting data from reports (e.g., piloting forms, done independently, in duplicate), any processes for obtaining and confirming data from investigators *	50 (12)	383 (88)	Data extraction form	169 (39)	264 (61)	/
			Data extraction process described	258 (60)	175 (40)	/
			Obtain missing data	76 (18)	357 (82)	/
12. Data items: List and define all variables for which data will be sought (e.g., PICO items, funding sources), any pre-planned data assumptions and simplifications	6 (1)	427 (99)	List of data for extraction *	219 (51)	214 (49)	/
			Variables defined *	29 (7)	404 (93)	/
			Any data assumptions reported	17 (4)	416 (96)	/
13. Outcomes and prioritisation: List and define all outcomes for which data will be sought, including prioritisation of main and additional outcomes, with rationale	3 (1)	430 (99)	Primary/main outcome(s) * specified as such	418 (97)	15 (3)	/
			Primary/main outcome(s) measure specified *	235 (54)	198 (46)	/
			Additional outcomes specified/ state None *	430 (99)	3 (1)	/
			Additional outcomes: measures specified *	131 (30)	180 (42)	122 (28)
			Rationale for choice of outcome(s)	8 (2)	425 (98)	/
14. Risk of bias in individual studies: Describe anticipated methods for assessing risk of bias of individual studies, including whether this will be done at the outcome or study level, or both; state how this information will be used in data synthesis *	41 (9)	392 (91)	No risk of bias assessment planned, and justification provided	4 (1)	3 (1)	426 (98)
			Risk of bias tools named for all study types included	362 (84)	67 (16)	4 (1)
			Outcome or study level or both	310 (71)	119 (28)	4 (1)
			Domains/outcomes for risk of bias assessment stated	342 (79)	87 (20)	4 (1)
			Risk of bias assessment process described	296 (68)	133 (31)	4 (1)
			How risk of bias findings will be used in the synthesis	64 (15)	365 (84)	4 (1)
15a. Synthesis: Describe criteria under which study data will be quantitatively synthesized	200 (46)	233 (54)	Criteria for doing a quantitative synthesis/ meta-analysis described *	131 (30)	233 (54)	69 (16)
15b. If data are appropriate for quantitative synthesis, describe planned summary measures, methods of handling data, and methods of combining data from studies, including any planned exploration of consistency (e.g., I ^2^, Kendall’s tau)	70 (16)	363 (84)	Summary measures *	202 (46)	163 (38)	68 (16)
			Statistical method *	89 (20)	276 (64)	68 (16)
			Use of fixed or random effects or both *	194 (44)	171 (40)	68 (16)
			Data handling: conversion to same format	106 (24)	259 (60)	68 (16)
			Data handling: missing data	14 (3)	351 (81)	68 (16)
			Combining data/ exploration of consistency	179 (41)	186 (43)	68 (16)
			Name of software to be used for meta-analysis	204 (47)	161 (37)	68 (16)
15c. Describe any proposed additional analyses (e.g., sensitivity or subgroup analyses, meta-regression)	84 (19)	349 (81)	Subgroup analyses planned: co-variants named *	344 (79)	21 (5)	68 (16)
			Methods for subgroup analyses reported	25 (6)	280 (65)	128 (29)
			Sensitivity analyses planned	85 (19)	280 (65)	68 (16)
15d. If quantitative synthesis is not appropriate, describe the type of summary planned *	227 (52)	206 (48)	Descriptive, narrative, or qualitative synthesis planned	194 (45)	55 (12)	184 (43)
			Descriptive, narrative or qualitative synthesis methods described	49 (11)	200 (46)	184 (43)
			Other analyses planned	3 (1)	11 (3)	419 (96)
16. Meta-bias(es): Specify any planned assessment of meta- bias(es) (e.g., publication bias across studies, selective reporting within studies)	72 (17)	361 (83)	Publication bias to be assessed	94 (21)	271 (63)	68 (16)
			Outcome reporting bias to be assessed	4 (1)	361 (83)	68 (16)
17. Confidence in cumulative evidence: Describe how the strength of the body of evidence will be assessed (e.g., GRADE)	37 (9)	396 (91)	Overall assessment of included studies planned	40 (9)	393 (91)	/
			Methods specified	38 (9)	395 (91)	/

* Item/element required in PROSPERO **Item/element identified in PROSPERO but as optional

The scores by PRISMA-P item and by breakdown of items are presented in Table 2. The full dataset with assessment outcomes and scores for individual records, and the subgroup analyses scoring are available on the OSF ( *Underlying data* ^16^).

### Scoring for 63 elements of the PRISMA-P items

The score for some of the 19 items was reduced as a result of just one or two of the constituent elements being omitted from reports while others were relatively regularly identified.

Although overall the review question (item 7) was not found to contain all the expected elements, most did specify the elements of population (397, 92%) and the intervention (416, 96%) and just over half included the outcomes (237, 55%). The comparator was less frequently included (142, 33%); this may have been because of the intention of the review but where this was clear, the item was scored as not applicable (6%).

Information sources (item 9) was scored as completed in only two records (1%) overall; however, for the individual elements 431 (99%) did name the electronic databases to be searched, 289 (67%) said whether they planned to search study registries, and 238 (55%) indicated search dates. In item 10, provision of a draft search strategy (91, 21%) or search terms (100, 23%) was poor; but restrictions such as to English language papers were reported in 332 (77%).

Reporting of item 13, outcomes, scored badly overall (3, 1%) as, although the outcomes were included in most records (Primary 418, 97%; Secondary 430, 99%) only 8 (2%) were assessed as having provided a rationale for their choice of outcomes. Similarly, in item 14, the absence of information on how the risk of bias would be used in the synthesis, detracted from the high rate of inclusion of risk of bias tools and use. Reporting of the details for a quantitative synthesis, item 15b, had one element with a very low score (handling missing data, 14, 3%), the other six elements scored between 89 (20%) and 204 (47%).

In three items, the overall score reflected the general picture from the included elements. In item 6, rationale, both the reason for undertaking the review and the context were infrequently identified. PRIMSA-P items 16, meta-bias(es) and 17, confidence in cumulative evidence, were rarely reported. Only context is classified as optional information in PROSPERO, the remainder of these elements are not explicitly requested.

There appears to be a trend towards higher frequency of reporting of elements that are mandatory in PROSPERO, for example, in the eligibility criteria (item 8) and risk of bias (item 14). The trend is also seen in item 13, the required specification of primary and secondary outcomes, both frequently reported, but with a drop in specifying measures, which was optional.

### Subgroup comparisons

The subgroup comparisons, which were all pre-defined, investigated the stage of review at registration; whether or not information was reported on source of funding, sponsorship or support and where none was indicated; and whether or not the relevant box in the registration form had been ticked to indicate a meta-analysis was planned.

There were no differences in total scores for the 19 PRISMA-P items or the 63 elements, between those records registered before screening against eligibility criteria had started and those records registered after screening had commenced. This held true for the mean, standard deviation, median and range of scores.

A 6% difference was seen in the total score achieved for the meta-analysis (23%) vs no meta-analysis (29%) groups in the assessment of the 19 PRISMA-P items. The difference was reduced to 2% when considering the breakdown of 63 elements within the reported items (52% vs 54%). At both item and element level, the group of records with no planned meta-analysis scored slightly higher, but with a higher standard deviation from the mean and wider range of scores achieved.

Across all results for both the 19 items and 63 elements, the group with funding, sponsorship or support, scored slightly higher than those not receiving funding, sponsorship or support.

The results of the subgroups investigated are presented in Table 3. The subgroup scores by individual PRISMA-P reporting item are available on the OSF ( *Underlying data* ^16^).

**Table 3.** Subgroup comparisons.

Subgroup	Variable	No. of records	Total possible score	Total score achieved N (%)	Mean score (SD)	Median score	Range of scores
For 63 PRISMA-P reporting elements							
**Stage of review at** **registration**	Before screening started	245	4655	1181 (25)	4.8 (1.9)	5	2–11
	After screening started	188	3572	900 (25)	4.8 (1.8)	4	2–10
**Meta-analysis planned**	M-A	250	4750	1088 (23)	4.4 (1.5)	4	2–9
	No M-A	183	3477	993 (29)	5.4 (2.1)	5	2–11
**Funded / Sponsored /** **Supported**	Funded etc.	381	7239	1841 (25)	4.8 (1.9)	4	2–11
	Not funded etc.	52	988	240 (24)	4.6 (1.6)	4	2–8
For 63 PRISMA-P reporting elements							
**Stage of review at** **registration**	Before screening started	245	15435	8214 (53)	33.5 (5.9)	33	18–47
	After screening started	188	11844	6255 (53)	33.3 (5.8)	33	21–47
**Meta-analysis**	M-A	250	15750	8244 (52)	33.0 (5.2)	32	21–45
	No M-A	183	11529	6225 (54)	34.0 (6.6)	34	18–47
**Funded / Sponsored /** **Supported**	Funded etc.	381	24003	12804 (53)	33.6 (5.9)	33	18–47
	Not funded etc.	52	3276	1665 (51)	32.0 (5.3)	31	22–46

We present the scores by the 19 PRISMA-P items and by the breakdown of 63 elements for the ten countries and topics with the highest number of assessed records, and for number of authors listed in Table 4. None of these factors appear to have a marked influence on the number of PRISMA-P items or elements reported in PROSPERO records.

**Table 4.** Overall scores by country, number of authors and topic of review.

	No of records	For the 19 PRISMA-P items assessed				For the 63 elements assessed			
		Overall score (% of possible score)	Mean score (SD)	Median score	Range of scores	Overall score (% of possible score)	Mean score (SD)	Median score	Range of scores
Country (10 with most assessed records)									
Australia	33	179 (28)	5.4 (2.1)	5	2–11	1115 (54)	33.8 (6.2)	32	21–47
Brazil	53	272 (27)	5.1 (1.9)	5	2–9	1826 (55)	34.5 (6.0)	35	18–46
Canada	37 *	197 (28)	5.3 (2.1)	5	2–9	1301 (56)	35.2 (6.7)	35	21–45
China	101	418 (22)	4.1 (1.3)	4	2–10	3385 (54)	33.5 (4.5)	34	23–45
England	46	259 (29)	5.6 (2.2)	5	2–10	1620 (55)	35.2 (6.9)	35.5	22–47
Germany	11 *	59 (28)	5.4 (2.3)	4	3–10	380 (55)	34.5 (6.2)	33	26–47
Italy	15	71 (27)	4.7 (1.8)	4	3–9	499 (57)	33.3 (6.2)	32	24–47
Netherlands	13	68 (28)	5.2 (2.1)	5	2–9	439 (53)	33.8 (7.0)	33	23–47
Spain	13	64 (26)	4.9 (1.8)	4	2–7	426 (52)	32.8 (5.6)	33	22–42
USA	48	242 (27)	5.0 (2.2)	4	2–10	1526 (51)	31.8 (6.4)	31	21–47
Number of authors									
0–3	202	956 (25)	4.7 (1.8)	4	2–10	6648 (52)	32.9 (5.9)	32	18–47
4–6	179	867 (25)	4.8 (1.9)	5	2–11	6008 (53)	33.6 (5.7)	34	21–47
7+	52	258 (27)	5.0 (1.9)	4	2–9	1813 (56)	34.9 (5.9)	34	21–47
Topic of review (10 with most assessed records)									
Cancer	42	184 (23)	4.4 (1.8)	4	2–10	1326 (50)	31.6 (5.6)	31	21–47
Cardiovascular	58 *	278 (25)	4.8 (1.8)	4	2–10	1952 (53)	33.7 (5.5)	33	21–46
Complementary therapies	43	211 (26)	4.9 (1.8)	5	2–9	1511 (56)	35.1 (6.0)	36	22–44
Endocrine and metabolic disorders	34 *	175 (27)	5.1 (2.1)	5	2–10	1204 (56)	35.4 (6.1)	36	21–47
Mental health and behavioural conditions	51	266 (27)	5.2 (2.0)	5	2–10	1762 (55)	34.5 (5.7)	33	21–44
Musculoskeletal	70	335 (25)	4.8 (2.0)	4	2–11	2295 (52)	32.8 (6.2)	32	18–47
Neurological	42 *	221 (28)	5.3 (1.9)	5	2–11	1443 (55)	34.4 (6.1)	33.5	23–47
Physiotherapy	36	174 (25)	4.8 (1.8)	4	2–8	1194 (53)	33.2 (5.8)	32.5	18–43
Rehabilitation	42 *	201 (25)	4.8 (2.1)	4	2–11	1393 (53)	33.2 (5.7)	32.5	23–47
Surgery	49	251 (27)	5.1 (1.8)	5	2–10	1644 (53)	33.6 (5.2)	33	23–47

*numbers differ from Table 1 because of the record(s) excluded at assessment

## Discussion

Publication and registration of a systematic review protocol provides transparency in the review process, allowing readers to see the efforts made to minimise biases and where biases may still have influenced the final review findings. There is empirical evidence that few of the protocol registrations in PROSPERO have a corresponding published report ^9^. Where there is no protocol, the registration provides the only public record of what was originally planned. This study set out to establish to what extent PROSPERO registrations of systematic review protocols of healthcare interventions reported on items in the PRISMA-P reporting guidelines.

Using a random sample of 433 PROSPERO records from 2018, two researchers independently assessed the frequency of reporting of 19 PRISMA-P items, with 63 individual elements. The results show that while some key methodological details are relatively frequently reported, much of the information recommended in PRISMA-P is missing. Reporting was unsurprisingly more frequent for items that are mandatory in PROSPERO than those that are optional. Comparisons by stage of review at registration, whether meta-analysis was planned and whether funding or sponsorship was reported showed no meaningful differences between groups. The slight difference between groups with a planned meta-analysis or none may be because in PRISMA-P more details are specified for the reporting of a meta-analysis than for a descriptive, narrative or qualitative analysis.

The review protocol is a detailed record of the planned methods developed through an iterative process ^5^. Once finalised or close to finalising, the key methodological details should be registered in PROSPERO ^8^. These are two separate but inter-related activities. PROSPERO was launched in 2011, a time when there were few opportunities to publish protocols, however, registration is not meant to be a substitute for preparation of a protocol. PROSPERO and PRISMA-P 2015 requirements are not aligned as they serve different purposes. However, a stated aim of registration is to facilitate comparison of what was planned with what is reported. Even if limited information were registered, we would expect the mandatory fields in PROSPERO to be fully completed. This was not the case, particularly for details related to outcome measures, assessment of risk of bias and quantitative analysis methods. It would not be reasonable to expect that PROSPERO records meet all the PRISMA-P recommended items, given the differences in purpose between a protocol and registration, but it is important to understand what information is available where registration is the only public source.

Eligibility criteria and type of analysis planned were most frequently reported and are all separate required fields in PROSPERO. However, study selection process, which is optional, was also a higher frequency reported item. This may be explained by considering that some elements of items, such as eligibility criteria, study selection and risk of bias have what might be considered a standard, recognisable format that facilitates reporting. Other items need a more nuanced approach underpinned by a clear understanding of systematic review methods, and therefore may be associated with being less frequently reported due to a lack of confidence or experience with these aspects of review methods. For example, how risk of bias will be used in the synthesis, data handling in a meta-analysis, meta-biases and confidence in cumulative evidence, all had low scores. Part of the problem may be the uncertainty of what the searches will find when designing a systematic review but needing to know so the design is appropriate. For example, the intention may be to perform a meta-analysis, this may not be possible once the studies for inclusion have been identified. While, both PROSPERO and PRISMA-P acknowledge that protocols are iterative documents and may need to be amended, changes should be documented, justified and the stage of review at the time of the amendment made clear. Therefore, it is better to record alternative options for activities such as how data will be analysed and the conditions for selection of option when finalising the protocol.

Differences in frequency of reporting may also reflect where researchers considered items to be less or more important than others. For example, naming the software used for data management may not be seen as crucial, whereas the eligibility criteria and approach to synthesis are.

There are strengths and limitations to this study. The assessed sample of 433 records was representative of all the eligible 2018 non-Cochrane intervention reviews registered in PROSPERO. As a result, the findings may reasonably be generalised to other registrations of healthcare interventions, but not necessarily other types of registered reviews excluded from our sample.

PRISMA-P is a reporting guideline and not a rating scale, so judgements about whether sufficient information had been provided for some items carried a degree of subjectivity. The assessment guide and form developed for the study aimed to maximise objectivity but in accordance with PRISMA-P did not weight importance of items. Although two researchers independently carried out the assessments, achieving an overall agreement rate of 90%, subjectivity was minimised but not eliminated.

PROSPERO was developed in 2011 to record key protocol details and does not necessarily accord with everything subsequently recommended in the 2015 PRISMA-P reporting guidelines. Some registration items are mandatory and others optional. However, this study looked at records that had no other protocol output and arguably should therefore have provided PRISMA-P level detail. The evidence that protocol details are only available in PROSPERO for around 96% of non-Cochrane reviews makes the infrequency of reporting of items a concern ^9, 10^. Based on the findings of other studies, promoting improved reporting of protocol details may help increase the quality of systematic reviews ^17, 18^.

Protocols are iterative documents and even after a review has started there may be legitimate reasons for amendments. Such changes should and can be reported in a registration record, with their justification and timing. Just over two thirds of PROSPERO records have more than one version ( Figure 1). While focussing on single entry records to be certain that any changes were not made after completion of the review this may have excluded records where more complete information was added to the record over time at key points in the review process.

This study simply looked at whether items were reported and not at the level of detail or suitability/appropriateness of the planned methods. Use of a scoring system giving equal weight to all items and elements as PRISMA-P does, is a limitation of this study because PROSPERO identifies information as either mandatory or optional. However, the scoring used in this study only relates to the presence or absence of information, and we have indicated the mandatory/optional fields in Table 2. The option of ‘partially reported’ could have been used at assessment but was avoided to minimise subjectivity. The focus was on simply establishing whether items were reported or not. The assessors focussed on whether the information was reported or could reasonably be inferred from what was reported. Assessing the quality of planned methods in protocol registrations needs to be the subject of further research.

This study shows that there is work to be done to promote the complete reporting of items recommended in the guidelines for systematic review protocols when the registration in PROSPERO is the only place they can be accessed. This is in line with other research that has identified issues with the quality of reporting, publication and outcome reporting biases in systematic review protocols in general ^3, 9, 11, 13, 19, 20^. As proposed in the PRISMA-P statement paper, actions and potential benefits to encourage adherence to PRISMA-P will take a joint effort on the part of a host of stakeholders, including reviewers, registries, and journal editors ^5, 21^.

## Conclusions

PROSPERO provides reviewers with the opportunity to be transparent in their planned methods and demonstrate efforts to reduce bias. However, where the PROSPERO record is the only available source of *a priori* reporting, there is a significant shortfall in the items reported, compared to those recommended in PRISMA-P. This presents peer reviewers and others wishing to assess the validity of the final review with challenges in interpretation. PROSPERO records are not peer reviewed or assessed for methodological quality, it is the responsibility of those registering their review to complete the registration form fully or provide access to a complete protocol. There are several areas requiring particular attention when completing the registration form. These include explaining the rationale for undertaking the review in the context of what is known; providing information sources beyond a list of databases to be searched; and reporting reproducible process methods for data management, study selection and risk of bias assessment. In addition, defining variables for data extraction, how specified outcomes will be measured, and the planned analyses, with criteria for undertaking a quantitative synthesis should all be included in detail.

This study only looked at whether recommended items were reported or not in PROSPERO records. Further research is needed to assess the quality of the planned methods in systematic review protocol registrations.

## Data availability

### Underlying data

Open Science Framework: PROSPERO and PRISMA-P, https://doi.org/10.17605/OSF.IO/7PW4G ^16^.

### Extended data

Open Science Framework: PROSPERO and PRISMA-P, https://doi.org/10.17605/OSF.IO/7PW4G ^16^.

This project contains the following underlying data:

- Study protocol
- Items, scoring options and guidance/rules for assessment of PROSPERO records compared to PRISMA-P reporting requirements

Data are available under the terms of the Creative Commons Attribution 4.0 International license (CC-BY 4.0).
