# Supplementary material for: An assessment of the extent to which the contents of PROSPERO records meet the systematic review protocol reporting items in PRISMA-P
Source: F1000Res. 2020 Sep 10;9:773. Originally published 2020 Jul 27. [Version 2] doi: 10.12688/f1000research.25181.2 (PMC7431973; doi:10.12688/f1000research.25181.2)
Supplement: Peer review history

# Peer review: Reviewer response for version 1

**DOI:** 10.5256/f1000research.27788.r68890

**Published:** 2020-08-17

**Authors:** Xin Sun

**Affiliations:** 1 Chinese Evidence-Based Medicine Center and Cochrane China Center, West China Hospital, Sichuan University, Chengdu, China

This study conducted a methodological survey to assess the extent to which the contents of PROSPERO records meet the systematic review protocol reporting items in PRISMA-P. This paper addresses an important research question, and the findings may have implications for the reporting of systematic review protocols. However, there are a few issues for authors to consider:

1. One aim of PRISMA-P is to aid authors in transitioning their systematic review protocols prepared in accordance with PRISMA-P into full text, while the authors used records from PROSPERO (i.e., not full-text) to assess the compliance to PRISMA-P reporting items, which may be a limitation that should be discussed in this paper.
2. In the methods part, it could be desirable that the authors could clearly report how the 17 numbered items of PRISMA-P were broken down into 63 elements.
3. The author should clearly report whether the subgroup analyses reported in table 3 were pre-planned.
4. The use of a scoring scheme for PRISMA-P and the 63 elements may not be optimal, given the potential difference in item importance, which should be added to the discussion part as a limitation.
5. In table 2, values in parentheses are percentages, which should be indicated in the table.

Is the work clearly and accurately presented and does it cite the current literature?

Yes

If applicable, is the statistical analysis and its interpretation appropriate?

Partly

Are all the source data underlying the results available to ensure full reproducibility?

Yes

Is the study design appropriate and is the work technically sound?

Yes

Are the conclusions drawn adequately supported by the results?

Yes

Are sufficient details of methods and analysis provided to allow replication by others?

Yes

Reviewer Expertise:

Clinical Epidemiology

I confirm that I have read this submission and believe that I have an appropriate level of expertise to confirm that it is of an acceptable scientific standard.

# Response

**Published:** 2020-09-01

**Authors:** Alison Booth

**Affiliations:** York Trials Unit, Department of Health Sciences, University of York, Heslington, York, UK

We thank you for your peer review and give our responses as follows:

*1. One aim of PRISMA-P is to aid authors in transitioning their systematic review protocols prepared in accordance with PRISMA-P into full text, while the authors used records from PROSPERO (i.e., not full-text) to assess the compliance to PRISMA-P reporting items, which may be a limitation that should be discussed in this paper.*

Thank you for raising this point. We agree and have addressed this point in the addition of the following to the discussion:

PROSPERO and PRISMA-P 2015 requirements are not aligned as they serve different purposes. However, a stated aim of registration is to facilitate comparison of what was planned with what is reported. Even if limited information were registered, we would expect the mandatory fields in PROSPERO to be fully completed. This was not the case, particularly for details related to outcome measures, assessment of risk of bias and quantitative analysis methods. It would not be reasonable to expect that PROSPERO records meet all the PRISMA-P recommended items, given the differences in purpose between a protocol and registration, but it is important to understand what information is available where registration is the only public source.

*2. In the methods part, it could be desirable that the authors could clearly report how the 17 numbered items of PRISMA-P were broken down into 63 elements.*

We have added an example from the study protocol to illustrate the description of how the elements were derived from the 19 PRISMA-P items, as follows:

Where the PRISMA-P description for an item specified more than one piece of information, the individual elements were listed as subsets of the items. For example, item 14. Risk of bias in individual studies, says: “Describe anticipated methods for assessing risk of bias of individual studies, including whether this will be done at the outcome or study level, or both; state how this information will be used in data synthesis.” Scoring for this item will be for each of the following separate elements: No risk of bias assessment planned and justification provided; Risk of bias tools named for all study types included; Outcome or study level or both; Domains/outcomes for risk of bias assessment stated; Risk of bias assessment process described; How risk of bias findings will be used in synthesis. Applying this approach to the 19 items resulted in a list containing 63 elements to be reported.

*3. The author should clearly report whether the subgroup analyses reported in table 3 were pre-planned.*

We can confirm they were all included in the study protocol, available at https://doi.org/10.17605/OSF.IO/7PW4G. We have added ‘which were all pre-defined’ to the section on Subgroup comparisons in the manuscript.

*4.The use of a scoring scheme for PRISMA-P and the 63 elements may not be optimal, given the potential difference in item importance, which should be added to the discussion part as a limitation.*

This is an important point thank you, which we have incorporated into the discussion as follows:

This study simply looked at whether items were reported and not at the level of detail or suitability/appropriateness of the planned methods. Use of a scoring system particularly as all items and elements carried the same weight is a limitation of this study. The scoring does not accord with the PROSPERO dataset which identifies information as either mandatory or optional. For this reason we have indicated the mandatory/optional fields in Table 2. The scoring only relates to the presence or absence of information. The option of ‘partially reported’ could have been used at assessment but was avoided to minimise subjectivity. The focus was on simply establishing whether items were reported or not. The assessors focussed on whether the information was reported or could reasonably be inferred from what was reported. Assessing the quality of planned methods in protocol registrations needs to be the subject of further research.

We have also amended how the mandatory/optional fields are indicated in Table 2 so the difference is clearer. 

*5. In table 2, values in parentheses are percentages, which should be indicated in the table.*

Apologies for this omission, this has been corrected in the revised version.

# Peer review: Reviewer response for version 1

**DOI:** 10.5256/f1000research.27788.r68072

**Published:** 2020-07-31

**Authors:** Dawid Pieper

**Affiliations:** 1 Faculty of Health, Department of Medicine, University Witten/Herdecke, Cologne, Germany

This article is an analysis of how PROSPERO records adhere to the PRISMA-P guideline. The Analysis is based on a random sample of 439 PROSPERO records published in 2018. The authors conclude that reporting in PROSPERO should be improved given the fact that the PROSPERO record is often the only available source of a priori reporting.

 The manuscript is methodologically sound and well written. What I think can be improved is the discussion. I wonder what is the implication of this study. Do the authors want to make the point that PROSPERO records should follow PRISMA-P? To the best of my knowledge PRISMA-P is even not mentioned in the PROSPERO guidance. If this would be the intention then wy not allign PROSPERO with the PRISMA-P items. I admit that PRISMA-P has been primarily designed for SRs of healthcare interventions, but most items are General and would be applicable to other review types as well. I do not want to make the point that this is a great idea, but it is somehow a logical question resulting from your manuscript and this should be mentioned in the discussion.

 Registries and protocols should be seen as different entities, and thus I think that a perfect result of all PROSPERO records meeting all PRISMA-P items cannot be what we aiming for. If this would be the case, this would probably dilute the difference between a PROSPERO record and a protocol.

Is the work clearly and accurately presented and does it cite the current literature?

Yes

If applicable, is the statistical analysis and its interpretation appropriate?

Not applicable

Are all the source data underlying the results available to ensure full reproducibility?

Yes

Is the study design appropriate and is the work technically sound?

Yes

Are the conclusions drawn adequately supported by the results?

Partly

Are sufficient details of methods and analysis provided to allow replication by others?

Yes

Reviewer Expertise:

Research methods, clinical epidemiology

I confirm that I have read this submission and believe that I have an appropriate level of expertise to confirm that it is of an acceptable scientific standard.

# Response

**Published:** 2020-09-01

**Authors:** Alison Booth

**Affiliations:** York Trials Unit, Department of Health Sciences, University of York, Heslington, York, UK

We thank you for your peer comments and agree this is an important point. We have added the following paragraph to the discussion section:

The review protocol is a detailed record of the planned methods developed through an iterative process. Once finalised or close to finalising, the key methodological details should be registered in PROSPERO. These are two separate but inter-related activities. PROSPERO was launched in 2011, a time when there were few opportunities to publish protocols, however, registration is not meant to be a substitute for preparation of a protocol. PROSPERO and PRISMA-P 2015 requirements are not aligned as they serve different purposes. However, a stated aim of registration is to facilitate comparison of what was planned with what is reported. Even if limited information were registered, we would expect the mandatory fields in PROSPERO to be fully completed. This was not the case, particularly for details related to outcome measures, assessment of risk of bias and quantitative analysis methods. It would not be reasonable to expect that PROSPERO records meet all the PRISMA-P recommended items, given the differences in purpose between a protocol and registration, but it is important to understand what information is available where registration is the only public source.
